# Evaluating the Diagnostic Accuracy of Xpert MTB/RIF Assay in Pulmonary Tuberculosis

**DOI:** 10.1371/journal.pone.0141011

**Published:** 2015-10-23

**Authors:** Surendra K Sharma, Mikashmi Kohli, Raj Narayan Yadav, Jigyasa Chaubey, Dinkar Bhasin, Vishnubhatla Sreenivas, Rohini Sharma, Binit K Singh

**Affiliations:** 1 Department of Internal Medicine, All India Institute of Medical Sciences, New Delhi, India; 2 Department of Biostatistics, All India Institute of Medical Sciences, New Delhi, India; National Institute for Infectious Diseases (L. Spallanzani), ITALY

## Abstract

Pulmonary tuberculosis still remains a major communicable disease worldwide. In 2013, 9 million people developed TB and 1.5 million people died from the disease. India constitutes 24% of the total TB burden. Early detection of TB cases is the key to successful treatment and reduction of disease transmission. Xpert MTB/RIF, an automated cartridge-based molecular technique detects *Mycobacterium tuberculosis* and rifampicin resistance within two hours has been endorsed by WHO for rapid diagnosis of TB. Our study is the first study from India with a large sample size to evaluate the performance of Xpert MTB/RIF assay in PTB samples. The test showed an overall sensitivity and specificity of 95.7% (430/449) and 99.3% (984/990) respectively. In smear negative-culture positive cases, the test had a sensitivity of 77.7%. The sensitivity and specificity for detecting rifampicin resistance was 94.5% and 97.7% respectively with respect to culture as reference standard. However, after resolving the discrepant samples with gene sequencing, the sensitivity and specificity rose to 99.0% and 99.3% respectively. Hence, while solid culture still forms the foundation of TB diagnosis, Xpert MTB/RIF proposes to be a strong first line diagnostic tool for pulmonary TB cases.

## Introduction

According to the WHO Tuberculosis report, 2014, there were 9 million incident cases of TB globally, and South–East Asia and Western Pacific regions contributed 58% to this burden of TB with India having a share of 24% of the global burden [1]. Pulmonary tuberculosis (PTB) continues to be an important cause of preventable mortality in both developing and developed nations,particularly in the setting of HIV infection. The cornerstone of TB control remains early diagnosis and treatment. However, the emergence of resistant strains, including multi-drug resistant (MDR) and extremely drug resistant (XDR) strains has posed a significant challenge. Though advances in drug therapy have been limited, TB control has greatly benefited from the advent of newer diagnostic tests including use of liquid culture media and nucleic acid amplification tests such as line probe assay and Xpert MTB/RIF. Rapid diagnosis of TB significantly decreases the lag time in initiation of treatment, thereby reducing transmission rates [2].

For several decades smear microscopy and conventional culture techniques have been the mainstay of diagnostic testing for pulmonary tuberculosis. While smear microscopy has poor sensitivity and issues related to quality control [3], conventional solid culture techniques have the limitation of long turnaround time of several weeks. Liquid culture techniques were developed for early detection of *Mtb* growth, but the mean turnaround time of 21 days is still long for a diagnostic test to be effective in curbing transmission [4]. Such delays in diagnosis increase morbidity and mortality, predispose to secondary resistance and cause transmission of resistant strains. Nucleic acid amplification tests (NAAT) such as in-house polymerase chain reaction (PCR) for TB and line probe assay were developed for rapid detection of TB and identification of drug resistance. However, the conventional in-house NAATs require well-trained technical staff and sophisticated equipments. Also, for these PCR, there are no validation studies done in large sample size. As the conventional NAATs have various steps from DNA isolation to amplification [5], there are also chances of cross-contamination from environmental factors or carry-over contamination from other samples.

Xpert MTB/RIF is an automated, heminested real-time PCR that detects MTB and tests every positive sample for rifampicin sensitivity using molecular beacons [6]. Thus, results for both, presence of MTB and rifampicin resistance, are available in less than 2 hours, in stark comparison to the turnaround time of conventional drug-sensitivity testing of 8–10 weeks. It is a cartridge based nucleic acid amplification test (CBNAAT) that does not have any specific pre-requites for its set-up and requires little technical training. Further, as the reagent used for processing is bactericidal and tubercle bacilli are inactivated in vitro, biosafety risks are eliminated, thus enabling its use as a rapid point-of-care diagnostic test.

The present study was done at an intermediate reference laboratory of the Revised National Tuberculosis Control Program (RNTCP) of India, located at a tertiary care hospital with the objective of evaluating the performance of Xpert MTB/RIF assay in diagnosing PTB and detecting resistance to rifampicin, taking culture as the gold standard for confirmed diagnosis and drug sensitivity.

## Material and Methods

### Participants

The study was approved by the ethics committee of All India Institute of Medical Sciences (AIIMS, New Delhi). Adult subjects with the clinical suspicion of PTB were included (from September 2012 to December 2014) and were either treatment naïve, or were on anti-TB treatment (ATT) for not more than two weeks. Patients who were on ATT for more than 2 weeks were excluded from the study. Samples from these patients were received and diagnosed in the Tuberculosis Laboratory (accredited Intermediate Reference Laboratory [IRL] for Delhi-NCR by MoH & FW, Govt. of India) of the Department of Internal Medicine, AIIMS, New Delhi which is a tertiary care referral centre and patients from various health care settings are referred here. A total of 1492 samples from 1406 patients were included in the study consecutively, and written informed consent was taken from study subjects. Six hundred fifty seven patients were on ATT for less than 2 weeks before the date of sample collection for diagnostic tests. Respiratory samples included sputum (n = 1141), endotracheal tube aspirate (n = 146), broncho-alveolar lavage (BAL) fluid (n = 128), induced sputum (n = 73) and bronchial washings (n = 4)

### Test Methods

The samples were subjected to Ziehl-Neelsen (ZN) staining, Xpert MTB/RIF (Cepheid, Sunnyvale, US) assay and culture inoculation. The technicians performing culture inoculation were blinded from Xpert MTB/RIF test results and vice-versa. Culture and Drug Susceptibility Testing (DST) on culture media was taken as the reference standard for MTB detection and rifampicin susceptibility respectively.

#### Xpert MTB/RIF

The Xpert MTB/RIF test was performed using the G4 version of cartridges as per the manufacturer’s instruction (Cepheid, Sunnyvale, CA). Unprocessed samples were used directly for performing the test and no frozen samples were used in the study. The samples were processed for Xpert MTB/RIF as per the manufacturer’s instructions.

#### Sample decontamination

The sample volume, which was left after doing Xpert MTB/RIF assay, was processed by the standard decontamination protocol, using NALC-NaOH method with the final NaOH concentration of 1%. Following centrifugation, the supernatant was discarded and the pellet was dissolved in 1–1.5 ml of phosphate buffer saline (PBS).

#### AFB Smear

Two smears were made for each sample. One slide for AFB smear was made directly from the sample while the other slide was made after the sample was decontaminated. After decontamination, the resuspended pellet in PBS was used to make smear on a glass slide and these slides were then stained using ZN staining method as per the standard protocol and then observed under the microscope [7].

#### Culture inoculation, incubation and drug susceptibility testing

The concentrated sample obtained after decontamination was inoculated for culture in BACTEC mycobacterium growth indicator tube (MGIT) for liquid culture [8] and on two slopes of Löwenstein-Jensen (LJ) solid medium [9]. Isolates were identified as *Mycobacterium tuberculosis (Mtb)* by their slow growth rate, colony morphology, inability to grow on L-J media containing *p*-nitrobenzoic acid (500 μg/ml), by niacin and catalase tests and also by immunochrommatographic test kit (SD MPT64TB Ag kit) for liquid culture. Any diagnostic sample that was detected as non-tuberculous mycobacterium (NTM) by culture method was considered as ‘non-TB’. Drug susceptibility testing (DST) was carried out on LJ media by economic variant of 1% proportion method as per the standard operating procedure of RNTCP, India [9]. Rifampicin susceptibility was tested at a concentration of 40 μg/ml. Any strain with 1% (critical proportion) of bacilli resistant to the drug rifampicin was classified as resistant to the drug.

#### Sequencing of rpoB gene discordant samples

DNA was isolated from culture isolates with Genolyse buffer (Hain life sciences). Amplification of 305bp band of *rpoB* gene was done using primer sequence described previously [10]. Sequencing of 81-bp rpoB gene was carried out with ABI prism 3130xl genetic analyser (Applied Biosystems) and BigDye Terminator v3.1 cycle sequencing kit (Applied Biosystems). The sequencing results were analyzed by using BioEdit software and alignment was done by using clustalW. Data obtained were compared with standard H37Rv sequence.

### Statistical Methods

Data were analysed using STATA statistical software version 12.1 (StataCorp LP, College Station, TX, USA). Sensitivity, specificity and negative and positive predictive values were calculated.

## Results

**Fig 1:**
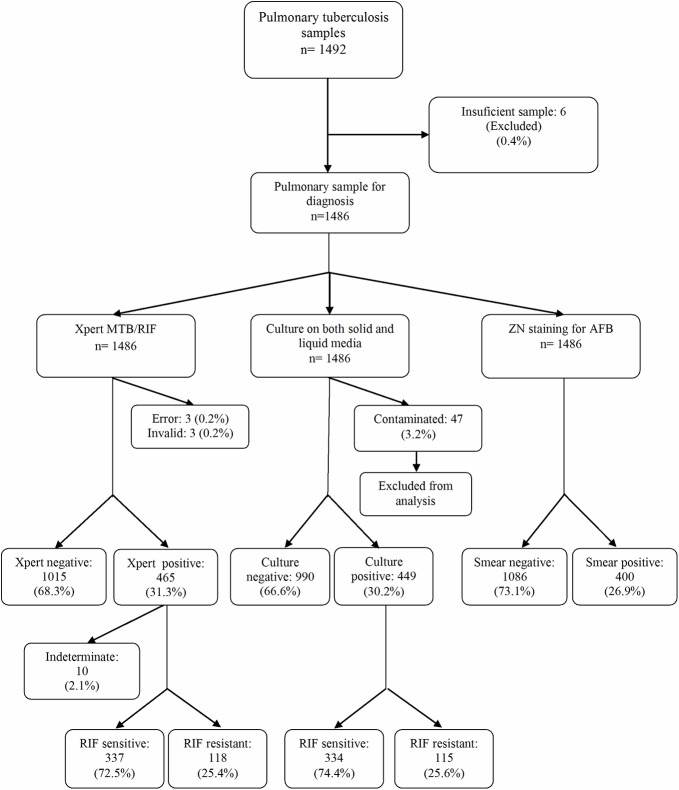
Study Design. *A total of 47 cultures were contaminated including three samples with invalid result by Xpert MTB/RIF and one sample with error by Xpert MTB/RIF. Two other samples which gave “error” by Xpert MTB/RIF were culture negative but were excluded from the study.

A total of 1406 patients were included in the study with mean age ± SD of 37.5 years ± 18.1 of which 909 were males and 497 were females. Of the 1492 samples in the study, 6 samples were insufficient in quantity for all three diagnostic modalities, and were hence excluded. Forty-nine samples were excluded from analysis because either the Xpert MTB/RIF assay gave the result as “error” or “invalid” or the samples were contaminated by culture. This left a total of 1437 samples to be included for analysis in the study (Fig 1).

The overall sensitivity of Xpert MTB/RIF in detecting culture positive pulmonary TB was 95.7% (430/449) and the specificity for excluding PTB was 99.3% (984/990). The sensitivity of Xpert MTB/RIF for detecting smear-negative culture-positive samples was 77.7% (56/72) and for detecting smear-positive culture-positive samples was 99.2% (374/377) [Table 1].

**Table 1:** Overall sensitivity and specificity of Xpert MTB/RIF assay for pulmonary samples.

	Sensitivity % (95% CI)			Specificity % (95% CI)	PPV % (95% CI)	NPV % (95% CI)
N = 1437	All Culture positive	Smear negative-culture positive	Smear positive-culture positive			
	95.7 (93.4–97.2)	77.7 (66.9–85.8)	99.2 (97.6–99.7)	99.6 (98.9–99.8)	99.0 (97.6–99.6)	98.1 (97.0–98.7)

PPV-Positive predictive value, NPV- Negative predictive value

There were 29 samples, which were contaminated by culture and were smear negative. Of these 29 samples, Xpert MTB/RIF detected *Mtb* in 14 samples.

The diagnostic performance of the assay varied in expectorated sputum, BAL fluids, induced sputum, endotracheal tube aspirate (ETA) and bronchial washing. The sensitivity for expectorated sputum, ETA, BAL, induced sputum, and bronchial washing were 96.9% (380/392), 87.5% (14/16), 90% (18/20), 84.2% (16/19), and 100% (2/2) respectively. High specificity was observed for all these samples. (Table 2)

**Table 2:** Diagnostic performance of Xpert MTB/RIF assay in different respiratory samples.

Sample type	Expectorated sputum [n = 1092]	Endotracheal tube aspirate [n = 143]	Bronchoalveolar lavage [n = 127]	Induced sputum [n = 71]	Bronchial wash [n = 4]
Sensitivity %	96.9 (94.7–98.2)	87.5 (63.9–96.5)	90.0 (69.9–97.2)	84.2 (62.4–94.4)	100 (34.2–100)
Specificity %	99.8 (99.2–99.9)	98.4 (94.4–99.5)	100 (96.5–100)	98.0 (89.0–99.6)	100 (34.2–100)
PPV %	99.7 (98.5–99.9)	87.5 (63.9–96.5)	100 (82.4–100)	94.1 (73.0–98.9)	100 (34.2–100)
NPV %	98.3 (97.0–99.0)	98.4 (94.4–99.5)	98.1 (93.5–99.5)	94.4 (84.8–98.0)	100 (34.2–100)

PPV-Positive predictive value, NPV- Negative predictive value. Values in parantheses are 95% confidence intervals

Results for molecular/genotypic DST for rifampicin as given by Xpert MTB/RIF were compared with the phenotypic DST done on solid media. Of the 430 Xpert and culture positive samples, 8 samples were indeterminate for RIF resistance by Xpert MTB/RIF assay and had to be excluded from the analysis. However, when these 8 samples were put for DST on solid media, 2 were RIF resistant and 6 were RIF sensitive. Out of the remaining 422 samples, 110 were resistant for rifampin by phenotypic DST of which 104 were resistant by Xpert MTB/RIF and six were sensitive for rifampin. Three hundred and twelve samples were sensitive for RIF by DST, of which 305 were sensitive and seven were RIF resistant by Xpert MTB/RIF. These data account for sensitivity of 94.5% (104/110) and specificity of 97.7% (305/312) [Table 3].

**Table 3:** Rifampin susceptibility testing by Xpert MTB/RIF and phenotypic DST.

	Rif resistant by DST	RIF sensitive by DST	Total
**RIF resistant by Xpert**	104	7	111
**RIF sensitive by Xpert**	6	305	311
**Total**	110	312	422

Sensitivity**-** 94.5% (88.6–97.4)

Specificity**-** 97.7% (95.4–98.9)

Positive Predictive Value- 93.6% (87.5–96.9)

Negative Predictive Value- 98.0% (95.8–99.1)

Data are presented as whole numbers. RIF- Rifampin, DST- Drug susceptibility testing

There were 13 discrepant samples, six where only Xpert was resistant for RIF and seven where only culture was resistant for RIF. These were then analyzed using gene sequencing for rpoB gene of *Mtb* DNA. Of these 13 cases, four were resistant by both Xpert and gene sequencing but culture sensitive for RIF. Five cases were sensitive by Xpert and gene sequencing but resistant by culture (Table 4). One sample had mixed growth with both wild type and mutant strains and was excluded from this analysis.

**Table 4:** DST Results from LJ and discrepant samples by rpoB gene sequencing.

	Rif resistant by DST and discrepant samples by sequencing	RIF sensitive by DST and discrepant samples by sequencing	Total
**RIF resistant by Xpert**	108	2	110*
**RIF sensitive by Xpert**	1	310	311
**Total**	109	312	421

Sensitivity- 99.0% (94.9–99.8)

Specificty- 99.3% (97.6–99.8)

Positive Predictive Value- 98.1% (93.6–99.5)

Negative Predictive Value- 99.6% (98.2–99.9)

Data are presented as whole numbers. RIF- Rifampin, DST- Drug susceptibility testing

*One sample was excluded from the analysis as it gave mixed growth results from sequencing.

The samples that were resistant by sequencing, mutations were found in codons 516 and 531 only. The sensitivity and specificity of the RIF resistance after resolving the discrepant samples using gene sequencing was 99.0% (108/109) and 99.3% (310/312) respectively.

## Discussion

There have been studies from India with large sample size that have evaluated the performance of Xpert MTB/RIF in patients with extrapulmonary TB [11,12], however, this is the first Indian study with a large sample size to have studied the performance of Xpert MTB/RIF assay with respect to solid medium culture and DST as the gold standard in patients with PTB. The study results clearly show that Xpert MTB/RIF has high sensitivity of 95.7% and specificity of 99.3% for detecting MTB in pulmonary samples of patients with PTB. For detecting smear negative-culture positive cases, our study results show a sensitivity of 77.7% and specificity 99.3% respectively. This is an important finding especially in a high TB burden country like India as this test will help in rapid diagnosis of smear-negative TB cases which were earlier a challenge for the TB control programmes. The results of our study are comparable to those a recent meta-analysis which reported the pooled sensitivity of Xpert in smear positive-culture positive PTB as 98%, and a sensitivity was 67% and specificity 99% for smear negative TB [13]. In our study, the sensitivity of Xpert to detect RIF resistance in pulmonary samples positive for PTB was 94.5% and the specificity for excluding RIF resistance was 97.7%. These results are consistent with previously reported data for RIF resistance [6,13,14].

The diagnostic performance of Xpert MTB/RIF in expectorated sputum was exemplary with a sensitivity of 96.9% and was higher than the sensitivity for ET aspirate and BAL (87.5% and 90% respectively). The results of our study for these respiratory samples were consistent with previously conducted studies [15–19]. However, as the proportions of samples of bronchial washing and induced sputum were small, the diagnostic utility of the assay in these samples needs to be further studied.

In the present study a total of 3 specimens gave ‘error’ as a result by Xpert MTB/RIF corresponding to an error percentage of 0.2% (3/1486). The MTB/RIF assay for rifampicin resistance was indeterminate in 0.5% (8/1492) cases. These values are much lower than the culture contamination rate of 3.2% (47/1486). The error rate in the present study was quite less as compared to the error rates >5% which were obseved in the earlier versions of Xpert MTB/RIF cartridges. G4 cartridges were modified in terms of the modified Ct values for the probes used in the assay, modification of the probe sequence and fluidics of the assay to decrease the error rates [20]. Further, post hoc-analysis of the study data showed that Xpert MTB/RIF was able to detect 14 out of 29 cases where smear was negative and cultures were contaminated, of which three were RIF resistant by Xpert MTB/RIF. Given the high specificity of Xpert MTB/RIF (99%), these cases are less likely to be false-positive. This is a significant finding from our study, particularly in the setting of a national TB control program. In the absence of Xpert MTB/RIF such patients are likely to undergo repeat testing for sputum smear microscopy and culture resulting in an unnecessary delay in initiation of treatment.

When the 13 samples which were discrepant by genotypic and phentotypic DST for rifampicin were analysed using gene sequencing, the results were more concordant with Xpert MTB/RIF than with the phenotypic DST. Pooling the results of gene sequencing, the overall sensitivity and specificity of Xpert MTB/RIF for detecting RIF resistance rose from 94.5% to 99% and from 97.7% to 99.3% respectively. This is critical finding because if only phenotypic DST was done and the result was sensitive for RIF (but resistant by genotypic DST), patients would have been put on only first-line anti-tubercular therapy, resulting in disease progression and transmission of drug resistant strains. In contradistinction, if phenotypic DST was resistant for RIF (sensitive by genotypic DST), patients would have inappropriately received treatment for MDR-TB resulting in unwanted adverse events due to prolonged administration of second-line anti-TB drugs and increased cost of treatment.

In the present study, DST was done on solid culture which adds to its merits as previously published literature has shown that automated BACTEC MGIT 960 can miss out on strains with certain resistance conferring *rpoB* mutations that can only be detected by DST on LJ medium [21]. Therefore, comparison of Xpert MTB/RIF with MGIT is likely to decrease specificity of Xpert MTB/RIF. Further, all pulmonary samples in this study were used directly for performing Xpert MTB/RIF without processing, and no frozen samples were included in the study.

Line probe assay (LPA), another molecular diagnostic test for TB, has shown to have sensitivity greater than 95% and a specificity of 100% [22]. However, LPA is recommended only in smear positive cases, while Xpert MTB/RIF in our study showed a sensitivity of 77.7% for detecting TB in smear negative samples. Hence, Xpert MTB/RIF provides a significant diagnostic edge in smear negative cases, as treatment can be started immediately without waiting for culture results.

While Xpert MTB/RIF may be the foremost choice amongst all molecular diagnostic tests, it has its own limitations. Resistance to RIF is taken as a surrogate marker for MDR-TB, but certain strains may exhibit only mono-resistance to RIF that may not warrant full line MDR therapy, thus, leading to over-estimation of the MDR-TB cases. Likewise, a study from Mumbai, India demonstrated how specimens with rifampicin results reported as sensitive by GeneXpert could be resistant to isoniazid [23]. Other drawbacks of Xpert MTB/RIF are requirement of stable electrical power supply, temperature control and annual calibration of instrument.

Regardless of all these limitations, addition of Xpert MTB/RIF assay to the present set of diagnostic modalities for TB on account of its unambiguous, rapid results, and high sensitivity and specificity will facilitate early diagnosis.

There are certain limitations of our study. The study had no clinical follow-up of the patients which does not give a clinical reference for the samples tested by Xpert MTB/RIF and culture. Also, as the patients were included in the study irrespective of their HIV status, the HIV status was not recorded for all patients as for some patients the HIV status was unknown either due to patients declining to get tested for HIV or no clinical follow up with the test results. However, our study fulfills the objective which was to evaluate the diagnostic utitlity of Xpert MTB/RIF assay in PTB samples using culture as the reference standard.

To conclude, our study highlights that Xpert MTB/RIF has high sensitivity and specificity for diagnosis of both smear positive and smear negative PTB cases with high rates of detection of RIF resistance and greater concordance with gene sequencing for RIF resistance when compared with culture. Our findings are similar to those reported by studies previously done in other countries. In resource-limited settings and less accessible areas where establishing a sophisticated laboratory for culture and DST conforming to the prescribed biosafety levels is difficult, Xpert MTB/RIF provides a viable option. Widespread application of this assay can increase the case detection rates of both drug sensitive and MDR-TB, thereby facilitating early treatment decisions and curbing transmission.
